# Identification of a Novel Idiopathic Epilepsy Locus in Belgian Shepherd Dogs

**DOI:** 10.1371/journal.pone.0033549

**Published:** 2012-03-23

**Authors:** Eija H. Seppälä, Lotta L. E. Koskinen, Christina H. Gulløv, Päivi Jokinen, Peter Karlskov-Mortensen, Luciana Bergamasco, Izabella Baranowska Körberg, Sigitas Cizinauskas, Anita M. Oberbauer, Mette Berendt, Merete Fredholm, Hannes Lohi

**Affiliations:** 1 Research Programs Unit, Molecular Medicine, University of Helsinki, Helsinki, Finland; 2 Department of Veterinary Biosciences and Department of Medical Genetics, University of Helsinki, Helsinki, Finland; 3 Folkhälsan Institute of Genetics, Helsinki, Finland; 4 Department of Small Animal Clinical Sciences, University of Copenhagen, Copenhagen, Denmark; 5 Department of Basic Animal and Veterinary Sciences, University of Copenhagen, Copenhagen, Denmark; 6 College of Veterinary Medicine, Kansas State University, Manhattan, Kansas, United States of America; 7 Department of Animal Breeding and Genetics, Swedish University of Agricultural Sciences, Uppsala, Sweden; 8 Referral Animal Neurology Hospital Aisti, Vantaa, Finland; 9 Department of Animal Science, University of California Davis, Davis, California, United States of America; National Cancer Institute, United States of America

## Abstract

Epilepsy is the most common neurological disorder in dogs, with an incidence ranging from 0.5% to up to 20% in particular breeds. Canine epilepsy can be etiologically defined as idiopathic or symptomatic. Epileptic seizures may be classified as focal with or without secondary generalization, or as primary generalized. Nine genes have been identified for symptomatic (storage diseases) and one for idiopathic epilepsy in different breeds. However, the genetic background of common canine epilepsies remains unknown. We have studied the clinical and genetic background of epilepsy in Belgian Shepherds. We collected 159 cases and 148 controls and confirmed the presence of epilepsy through epilepsy questionnaires and clinical examinations. The MRI was normal while interictal EEG revealed abnormalities and variable foci in the clinically examined affected dogs. A genome-wide association study using Affymetrix 50K SNP arrays in 40 cases and 44 controls mapped the epilepsy locus on CFA37, which was replicated in an independent cohort (81 cases and 88 controls; combined p = 9.70×10^−10^, OR = 3.3). Fine mapping study defined a ∼1 Mb region including 12 genes of which none are known epilepsy genes or encode ion channels. Exonic sequencing was performed for two candidate genes, *KLF7* and *ADAM23*. No variation was found in *KLF7* but a highly-associated non-synonymous variant, G1203A (R387H) was present in the *ADAM23* gene (p = 3.7×10^−8^, OR = 3.9 for homozygosity). Homozygosity for a two-SNP haplotype within the *ADAM23* gene conferred the highest risk for epilepsy (p = 6.28×10^−11^, OR = 7.4). ADAM23 interacts with known epilepsy proteins LGI1 and LGI2. However, our data suggests that the *ADAM23* variant is a polymorphism and we have initiated a targeted re-sequencing study across the locus to identify the causative mutation. It would establish the affected breed as a novel therapeutic model, help to develop a DNA test for breeding purposes and introduce a novel candidate gene for human idiopathic epilepsies.

## Introduction

Epilepsy is one of the most common neurological diseases affecting 1–3% of the human population [1]. Epilepsy refers to a group of chronic neurological symptoms characterized by recurrent unprovoked seizures. Seizures are transient symptoms of abnormal, excessive or synchronous neuronal activity in the brain and can be classified into two major types: focal-onset and primarily generalized. In focal-onset seizures, the synchronized activity is restricted to a single part of the cortex, and may or may not subsequently spread to recruit the thalamocortical pathways and result in secondary generalization. Focal motor seizures may be characterized by elementary motor events, which consist of a single type of stereotyped contraction of a muscle or group of muscles or by autonomic features or paroxysms of behavioral signs probably corresponding to disturbance of higher cerebral activity in humans known as psychic seizures [2]. In generalized seizures, the thalamocortical circuitry is involved in the attack and results in synchronized firing of neurons brain-wide, unconsciousness and often tonic-clonic seizures. In humans, epileptic syndromes are defined by such phenotypic criteria as age of onset, survival, type of electroencephalographic (EEG) abnormalities, seizure characteristics, and the type of stimulus that induces seizures [3]–[7]. A majority of epilepsies have a suspected polygenic background. However, only a few risk genes are known to date, and a large number of genes contributing to human epilepsy still remain to be identified [8].

Epilepsy is also the most common chronic neurological disorder in dogs, and has been identified by breeders as one of the top three diseases of concern. Canine epilepsy can be classified either as idiopathic (genetic) or symptomatic (structural/metabolic) according to recent ILAE recommendations in humans [9]. Epileptic seizures are classified according to initial clinical signs either as focal or generalized seizures [9], [10]. Additionally, focal seizures may become secondarily generalized.

The prevalence of epilepsy in purebred dogs is estimated to range from 0.5% to 1%. However, in some breeds there is a strong suspicion of an underlying genetic factor as there is an accumulation of epileptic individuals within families with an incidence as high as 20% [4], [11]–[18]. Moreover, the majority of the pedigree studies suggest a polygenic mode of inheritance.

Genetic homogeneity within dog breeds and heterogeneity across breeds, together with the recent advent of genome-wide mapping tools with high resolution provide a powerful approach for gene mapping of both simple and complex traits in dogs [19]–[22]. Naturally occurring spontaneous canine epilepsies resemble clinically human epilepsies and provide exciting models to further understand the genetics and the etiopathologies of seizure disorders. The first canine symptomatic epilepsy gene, *NHLRC1*, was found in the Miniature Wirehaired Dachshund presenting canine Lafora disease [23]. This was followed by eight genes related to particular forms of neuronal ceroid lipofuscinoses (NCLs) [24]–[31]. Most of these known canine progressive myoclonus epilepsy (PME) genes are orthologues of the corresponding human syndromes and two new NCL candidate genes, *ARSG* and *ATP13A2*, have been identified for human NCLs [24], [27].

The first canine IE mutation in the *LGI2* gene was recently identified in Lagotto Romagnolo dogs with focal remitting juvenile epilepsy [32], [33]. Despite efforts using either candidate gene [34] or low-resolution genome wide approaches [35], [36], the genetic background of many focal and generalized epilepsies remains largely unknown.

As part of our larger ongoing program to tackle the genetics of canine epilepsies (www.eurolupa.org), we have developed further resources to map the IE genes in Belgian Shepherds (BS) suffering from epilepsy dominated by focal seizures with or without secondary generalization [18]. Epileptic seizures vary from mild to intractable and typically have an onset around 3 years of age in this breed [18], [37]. Various pedigree analyses have suggested different modes of inheritance from simple recessive to polygenic with a major gene or a gene with incomplete penetrance [7], [18], [35], [36]. A recent microsatellite-based genome wide linkage scan with 366 dogs including 74 cases identified six tentative loci on four chromosomes, although none of them reached a genome-wide significance [36]. This could indicate genetic or phenotypic heterogeneity of epilepsy in BS. However, due to lack of power and resolution these results are not conclusive.

We have performed clinical characterizations including EEG recordings and a high-resolution genome-wide association study (GWAS) in a case-control cohort of BS dogs to identify IE loci. We successfully mapped a locus at CFA37 and defined a ∼1 Mb region containing novel candidate IE genes. This study establishes the first locus for the most common forms of seizures in dogs.

## Results

### Summary of the epilepsy cases collected for the investigation

To identify the genetic cause of IE in Belgian Shepherds (BS) we collected altogether 307 samples including 159 cases and 148 controls collected in Finland, Denmark and USA. Characterization of the Finnish study cohort was based on clinical examination of selected dogs and analysis of the owner-filled epilepsy questionnaires. To describe the Finnish cohort we analyzed 94 questionnaires from epileptic dogs as summarized in the **Table S1**. The vast majority of the dogs with only questionnaire data (78%) had also been diagnosed with epilepsy by a practicing veterinarian. The epileptic dogs showed a highly variable age of onset ranging from 3 months to 9 years with a mean at 3.3 years. The median seizure frequency was 5.25 per year with some dogs having less than one seizure per year and others having up to 10 seizures per day. The epileptic dogs had experienced on average 10 seizures (range 2–100) and one third presented clustered seizures (more than one seizure in a day). The typical duration of seizure was 2–4 minutes although ranging from 0.5–60 min. Almost half of the owners (42.7%) were able to identify phenomenology preceding convulsions as a sign of focal seizure activity. The typical clinical signs included restlessness, seeking of the owner's attention, drooling and nausea, which suggest a focal onset. Secondary generalization of focal seizures was commonly characterized by stiffening of limbs and neck, muscle fasciculation, tremor, drooling, staring, falling, tonic-clonic convulsions and urination. One third of the dogs did not react to owners' calls indicating a severely impaired consciousness. Postictal recovery time varied from minutes to hours.

The seizure types of the dogs were defined based on the seizure description. The majority of the dogs (37%) showed focal seizures with secondary generalization, one third of the dogs (34%) showed generalized seizures with unknown onset, 18% showed primarily generalized seizures and 7% of the dogs seizures were focal without secondary generalization. The seizure type of three dogs remained unclassified.

In the Finnish study cohort, 48 dogs out of 94 dogs (51%) were on anti-epileptic medication. Based on the 33 response reports, anti-epileptic medication was effective and prevented seizures in 18 (55%), halved the frequency in 10 (30%) and decreased it in 4 dogs (12%). Only one dog (3%) did not respond to medication. Seizure medication consisted mainly of Phenobarbital (88%) or potassium bromide (10%).

### Clinical studies

**Figure 1:**
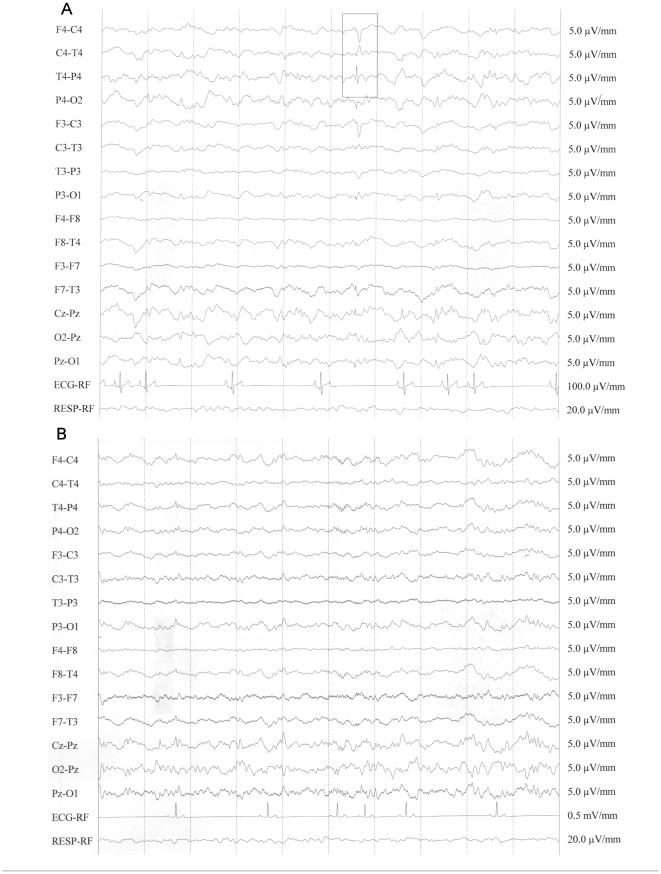
An example of interictal EEG recording for an epileptic (A) and for a healthy (B) BS dog. The epileptic and healthy dogs correspond to dogs 26 and 4C in Table 1, respectively. The Epileptic dog shows spike and slow waves in right central and posterior derivations. The control dog exhibits a high-voltage low-frequency background activity. Background activity is superimposed with focal beta bursts in frontal derivation. The EEG pattern is consistent with the sedation protocol used. Bipolar montage, time constant = 0.3 s; high filter 70 Hz; notch filter inserted.

Clinical examinations were performed on 17 Finnish cases and 4 controls (**Table 1**). All examined dogs were normal with regards to neurological examination, MRI, blood and CSF tests. These results exclude possible external causes of epilepsy and further support the presence of IE in the breed. At the visual examination of the EEG recordings, all dogs exhibited high-voltage low-frequency background activity. Background activity was superimposed with spindles or focal beta bursts in control dogs and in 2 dogs with epilepsy (dog 4 and dog 9). The standard descriptions used in human neurophysiology were adapted to describe all the EEG patterns [38]. Paroxysmal activity was observed in epileptic dogs, and it was characterized by sharp waves, spikes, and spike-and-slow-wave complexes in variable derivations (**Table 1** **,** **Fig. 1**). Two of the dogs exhibited midline spikes (dog 30 and dog 33), one had volley of sharp waves in centro-temporal-posterior right derivations (dog 9) and the other epileptic dogs exhibited spikes and spike-and-wave complexes in variable derivations. BETS and sleep spindles in healthy and epileptic dogs under medetomidine sedation were described previously [39]. Beta bursts are very similar to sleep spindles. They differ in having higher frequency, longer duration, and do not begin and end abruptly [40]. Both of these transients occur in the frontal, central, and parietal derivations. In humans, midline spike is supposed to represent epileptiform activity of uncertain clinical relevance [41], [42]. Spikes and spike-and-slow-wave complexes are considered as specific findings in many human epileptic syndromes [42], [43]. These findings were the only interictal abnormal EEG patterns detected in the dogs with epilepsy, suggesting a variable focal paroxysmal discharge.

**Table 1:** Summary of clinical examinations performed on 17 epileptic and 4 healthy Finnish BS dogs.

Dog ID	variation/sex	age	age of seizure onset	overall clinical examination	neurologic examination	blood chemistry	MRI	CSF	EEG activity	Regions where EEG activity were detected	Seizure type based on questionnaire^1^	diagnosis^2^
Dog 4	ter/female	13 y	2 y	norm	norm	norm	norm	norm	focal	Left anterior derivations	CFG	IE
Dog 6	gro/male	6 y	5,5 y	norm	norm	norm	norm	norm	NA		CFG	IE
Dog 7	gro/female	4 y	3 y	norm	norm	norm	norm	norm	NA		CFG	IE
Dog 8	gro/male	6 y	2 y	norm	norm	norm	norm	norm	NA		CF	IE
Dog 9	gro/female	7 y	3 y	norm	norm	norm	norm	norm	focal	Central (entire right hemisphere)	CFG	IE
Dog 11	gro/female	8 y	7 y	norm	norm	norm	norm	norm	NA		CFG	IE
Dog 12	gro/female	6 y	5 y	norm	norm	norm	norm	norm	NA		CFG	IE
Dog 13	gro/female	4 y	3 y	norm	norm	norm	norm	norm	focal	Right temporal posterior derivations	CFG	IE
Dog 17	ter/male	5 y	2,5 y	norm	norm	norm	norm	norm	NA		CFG	IE
Dog 18	ter/female	4 y	0,5 y	norm	norm	norm	norm	norm	NA		CFG	IE
Dog 20	ter/male	8 y	5 y	norm	norm	norm	norm	norm	NA		GUO	IE
Dog 22	ter/male	3 y	2,5 y	norm	norm	norm	norm	norm	focal	Right and left posterior derivations	CFG	IE
Dog 26	ter/female	3 y	2 y	norm	norm	norm	norm	norm	focal	Right central and posterior derivations	CFG	IE
Dog 27	ter/male	3 y	2 y	norm	norm	norm	norm	norm	NA		CFG	IE
Dog 30	ter/female	4 y	2,5 y	norm	norm	norm	norm	norm	focal	Midline	CF	IE
Dog 33	gro/female	5 y	1,5 y	norm	norm	norm	norm	norm	focal	Midline	CFG	IE
Dog 34	gro/male	6 y	3,5 y	norm	norm	norm	norm	norm	NA		NA	IE
Dog 1C	ter/female	3,8 y		norm	norm	norm	norm	norm	NA			healthy
Dog 2C	ter/male	6,9 y		norm	norm	norm	norm	norm	NA			healthy
Dog 3C	mal/female	7,5 y		norm	norm	norm	norm	norm	NA			healthy
Dog 4C	gro/female	6,3 y		norm	norm	norm	norm	norm	norm			healthy

1 CFG = complex focal generalized, CF = complex focal, GUO = generalized with unknown onset, NA = not available.

2 IE = idiopathic epilepsy.

The Danish epilepsy cases were investigated with interview questionnaires, clinical and neurological examination and para-clinical tests and has been characterized with respect to clinical epilepsy phenotype and semiology reported in previous publications [7], [18]. In general the clinical epilepsy phenotype displayed by the Finnish and the Danish cohorts were similar.

### GWAS, replication and fine mapping

**Figure 2:**
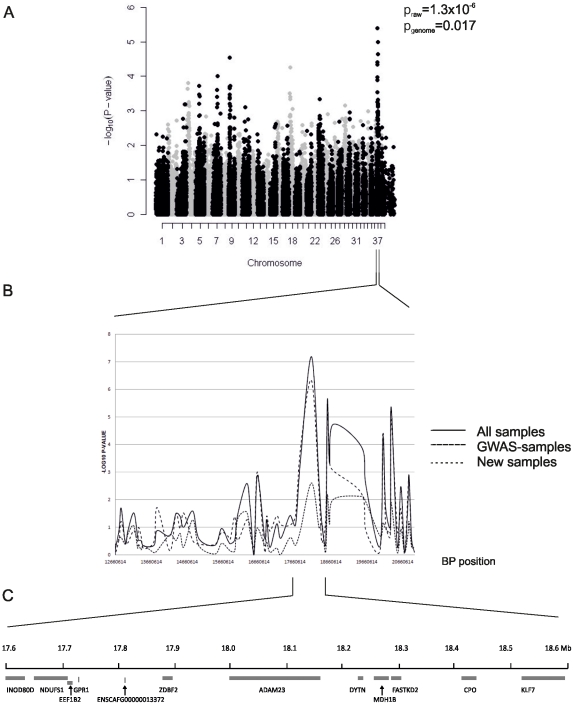
A genome-wide association study reveals a locus at CFA37. Genomic control –adjusted p-values are shown (A). Fine-mapping of a 8.3 Mb region with 96 additional SNPs on chromosome 37 defines a 1 Mb associated region (B). The region showing strongest association with epilepsy (17,525,804–18,623,591 bp) contains 12 genes including two neuronal candidates, *ADAM23* and *KLF7* (C).

To map the epilepsy genes we performed a GWAS with 40 cases and 44 seizure-free controls (27 cases and 27 controls from Finland and 13 cases and 17 controls from Denmark). The controls were >7 years old and country- and variant-matched to the cases. A significant association was detected around SNPs on CFA37 with the best SNP BICF2P890779 at 18,123,961 bp (p_raw_ = 1.3×10^−6^, p_genome_ = 0.017) (**Fig. 2A**). Other putative loci were found on chromosomes 3, 4, 9, 18, and 23 although not at a genome-wide significant level (**Fig. 2A**).

As a replication study we genotyped the SNP BICF2P890779 showing the strongest association with IE at CFA37 in an independent sample cohort of 81 cases and 88 controls (p = 3.7×10^−5^, OR = 2.6, 95% CI 1.6–4.1). A combined analysis of all genotyped samples including the original GWAS and the replication cohorts yielded a p-value of 9.7×10^−10^ and OR = 3.3 (95% CI 2.2–4.9) (**Table 2**). Homozygosity with respect to the allele A increased the risk of epilepsy by 5.4-fold (95% CI 3.1–9.3, p = 6.8×10^−10^). The frequency of the AA genotype was 0.62 among cases and 0.24 among controls. The nominal association signals on chromosomes 3, 4, 9, 18, and 23 were followed up by replication of the best-associated SNPs in each locus using 54 cases and 62 controls. None of these loci showed evidence for association in the replication cohort (**Table 2**).

**Table 2:** The summary of the results in GWAS, replication and combined datasets.

			GWA^1^						Replication^2^				Combined^3^			
Chromosome	SNP	Position	F_A^4^	F_U^5^	P_raw_	P_GC_	P_genome_	OR	F_A^4^	F_U^5^	P_raw_	OR	F_A^4^	F_U^5^	P_raw_	OR
3	BICF2P397912	82766251	0.60	0.33	3.91E-4	0.001	0.96	3.11	0.49	0.40	0.14	1.41	0.51	0.39	0.014	1.65
4	TIGRP2P58276	13088720	0.43	0.16	1.39E-4	5.25E-4	0.76	3.91	0.39	0.29	0.07	1.55	0.40	0.26	0.004	1.86
9	BICF2P1288768	18597315	0.43	0.47	4.71E-4	0.001	0.98	0.31	0.43	0.47	0.53	0.86	0.38	0.47	0.078	0.69
18	TIGRP2P239410	15846321	0.15	0.47	1.68E-5	8.95E-5	0.18	0.21	0.30	0.39	0.10	0.67	0.27	0.43	0.001	0.49
23	BICF2G630382382	22138085	0.20	0.48	1.60E-4	5.91E-4	0.79	0.27	0.25	0.31	0.22	0.73	0.24	0.37	0.005	0.53
37	BICF2P890779	18123961	0.53	0.18	1.34E-6	1.09E-5	0.02	5.41	0.72	0.50	3.72E-05	2.59	0.76	0.49	9.70E-10	3.29

1 GWAS dataset: 40 cases, 44 controls.

2 Replication dataset: chromosomes 3, 4, 9, 18, 23: 54 cases, 62 controls; chromosome 37: 81 cases, 88 controls.

3 Combined dataset: chromosomes 3, 4, 9, 18, 23: 94 cases, 106 controls; chromosome 37: 116 cases, 130 controls.

4 Frequency of the minor allele (based on GWAS controls) in affected individuals.

5 Frequency of the minor allele (based on GWAS controls) in unaffected individuals.

To fine map the associated locus we genotyped 83 BS cases and 99 BS controls with 96 SNPs from a 8.3 Mb region at CFA37 (12,660,614–20,989,289 bp). Fine mapping defined a ∼1 Mb region with the strongest association to a SNP BICF2P890779 at 18,123,961 bp (p_raw_ = 6.6×10^−8^, p_1000×perm_ = 1.0×10^−3^) (**Fig. 2B**).

### Candidate gene sequencing

The associated 1 Mb region at CFA37 contains 12 genes of which two, *ADAM23* and *KLF7*, have functions in neuronal systems (**Fig. 2C**). Mutations in *ADAM23* have not been found in epileptic patients, but it interacts with *LGI1*, a gene associated with familial temporal lobe epilepsy-1 (ETL1) in human [44] and with *LGI2*, which is the causative gene for benign focal epilepsy in dogs [33]. KLF7 is a neuronal transcription factor, which is required for neuronal morphogenesis and axon guidance in selected regions of the brain [45]. Sequence analysis of *KLF7* did not reveal any coding variants. Screening of the *ADAM23* exons revealed a non-synonymous variant in exon 12 (G1203A according to predicted mRNA XM_844759) at 18,113,688 bp causing an amino acid change (R387H according to XP_849852.1) in four affected dogs. To further investigate the frequency of the variant, we genotyped a total of 159 cases and 148 controls. The risk allele A frequency was 72% in the cases compared to 49% in the controls (p = 3.1×10^−9^, OR = 2.7, 95% CI: 1.9–3.8). Homozygosity for the A allele increased the risk (p = 3.7×10^−8^, OR = 3.9, 95% CI: 2.4–6.4). However, 22% of controls were also homozygous. Comparison of frequency of the R387H variant in different epilepsy types did not show enrichment to specific seizures (data not shown). This variation was also screened in three epileptic dogs from 38 other breeds (altogether 114 epileptic dogs), and we found that the homozygous AA genotype was present in some of the three affected dogs in altogether 12 breeds (19% of tested dogs) (Barbet, Beagle, Border Collie, Dachshund, Dalmatian, Golden Retriever, Irish Water Spaniel, Miniature Pinscher, Petit Basset Griffon Vendeen, Miniature Poodle, Rottweiler and Whippet). In 15 breeds, the risk allele was present in heterozygous form in some of the individuals (32%). Furthermore, Panther and PolyPhen-2 programs predicted that R387H is not pathogenic. Overall, these results suggest that the observed variation is not a causative mutation but rather a polymorphism, which is likely in the vicinity of the actual predisposing mutation. Indeed, we identified the highest risk of epilepsy among individuals homozygous for the haplotype composed of the risk-conferring alleles of the G1203A and BICF2P890779 variants (p = 6.28×10^−11^ OR = 7.4, 95% CI: 3.9–14.0) which are in strong linkage disequilibrium (LD) with each other (D′ = 0.87). This suggests that the functional variant lies within this haplotype block but is neither one of the two SNPs.

We investigated *ADAM23* as a candidate gene also through RNA expression studies. Expression level of *ADAM23* was compared between three healthy and three epileptic Belgian Shepherds from Denmark. All dogs were clinically examined. A difference in expression level between the two groups of dogs was not observed.

### Association of the CFA37 locus in other IE breeds

As part of our ongoing clinical and genetic studies on epilepsies, we have established well-characterized sample cohorts for many breeds presenting IE. To test whether the identified epilepsy locus at CFA37 associates with epilepsy in other breeds, we genotyped the best associated BS SNP (BICF2P890779) in an epilepsy cohort of 303 cases and 316 controls including samples from Lagotto Romagnolo, Miniature Pinscher, Kromfohrländer, Whippet, Border Terrier, Schipperke, Finnish Spitz and Finnish Lapphund (**Table 3**). Almost all tested Finnish Spitz and Schipperke dogs were homozygous for the A-allele and therefore no association could be calculated in these two breeds. Kromfohrländers (p = 0.003) and Whippets (p = 0.02) showed a tentative association (**Table 3**). These results in both breeds need to be confirmed in a larger sample cohort with additional markers before further conclusions.

**Table 3:** Association of *ADAM23* intronic SNP (BICF2P890779 at 18,123,961 bp) with epilepsy in 9 different breeds.

Breed	N cases+N controls	F_A^1^ (allele A)	F_U^2^ (allele A)	P	OR
Belgian Shepherd	116+130	0.76	0.49	**9.70E-10**	3.3
Kromfohrländer	23+18	0.93	0.67	**3.44E-03**	6.5
Whippet	24+26	0.81	0.60	**0.018**	2.9
Finnish Spitz	62+81	1.00	0.98	0.13	NA
Lagotto Romagnolo	23+23	0.35	0.48	0.20	0.6
Miniature Pinscher	22+20	0.89	0.80	0.27	2.0
Border Terrier	42+40	0.93	0.89	0.36	1.6
Schipperke	63+48	0.99	1.00	0.38	NA
Finnish Lapphund	44+60	0.79	0.82	0.58	0.8

1 Frequency of the minor allele (based on GWAS controls) in affected individuals.

2 Frequency of the minor allele (based on GWAS controls) in unaffected individuals.

Frequencies of allele A are shown for each breed. P-values<0.05 are bolded.

## Discussion

We describe here the second IE locus in dogs. A locus at CFA37 predisposes Belgian Shepherds to focal epilepsy with seizures originating from multiple cerebral lobes and without any detectable cerebral lesions on MRI studies. The first canine IE mutation was described in the Lagotto Romagnolos. This mutation causes a breed-specific focal epilepsy with remission [33], whereas BS dogs suffer from seizures that are commonly seen across breeds. Therefore our results may suggest a genetic locus for the most common forms of IE in dogs.

The clinically examined BS dogs had normal blood biochemistry, CSF, MRI and neurological examination and symptomatic epilepsy and seizures of extracranial origin was therefore not suspected. In the cases where interictal EEG was performed we detected paroxysmal activity originating from different cerebral lobes. The most common seizure type was a focal-onset with secondary generalization. The seizures in the minority of the dogs remained focal and some dogs had primarily generalized seizures. The fact that only half of the affected dogs received anti-epileptic medication suggests that epilepsy in the BS has a relatively mild course. Our study cohort included samples from several countries including a previously described cohort from Denmark [7], [18]. The onset and clinical features in different populations are similar. There are some differences in proportion of seizure types which may arise from the fact that a focal seizure onset may be challenging to observe and describe retrospectively by the owners. This is most likely the explanation why seizure distributions differ in the Danish and Finnish cohorts.

The CFA37 locus identified in this study is syntenic with the region on human chromosome 2q33 (206.8–208.2 Mb). Overlapping interstitial deletions in 2q24–31 have been described in many human epilepsies often associated with other developmental defects [46]. Our locus is close to these deletions but not syntenic. In addition, our clinical characterizations indicate that epileptic BS dogs present only a seizure disorder without developmental abnormalities. Another type of human epilepsy called familial partial epilepsy with variable foci (FPEVF) has also been mapped to 2q [47]. FPEVF is an autosomal dominant epilepsy with incomplete penetrance and characterized by epileptic seizures originating from different cerebral lobes [47], [48]. Affected individuals respond well to antiepileptic drugs and have no brain lesions. Causative mutations have not been found for FPEVF [47]–[51]. Although the clinical features in our dogs resemble the characteristics of human FPEVF, the most significant region in dogs is ∼10 Mb from the strongest association signal in human patients. Besides human, an overlapping epilepsy region has been found in WAG/Rij rats representing a model for human childhood absence epilepsy [52]. However, the syntenic region in rats covers an extensive region of the chromosome with many possible candidate genes.

Previous epidemiological studies have demonstrated a high prevalence of IE in the BS breed and various inheritance models have been suggested [7], [18], [35]–[37]. We found a major locus at CFA37 overlapping a previous tentative QTL [36], but with only a modest disease risk suggesting that still other susceptibility loci exist. Alternatively, the mildest focal seizures may have been missed and it is therefore possible that some controls are actually cases, which would result in the underestimation of the disease risk. This assumption is supported by a genealogical study performed in an extended family of dogs investigated over several years [7]. Our GWAS was performed in a relatively small sample cohort and with the older version of SNP chip arrays including ∼50,000 markers. It is possible that additional loci could be discovered with a larger sample size and higher resolution available today.

The 1 Mb region showing the strongest association includes 12 genes of which none encode ion channels commonly mutated in human IEs or other known epilepsy genes [53]. We screened two genes, *ADAM23* and *KLF7* for coding and splice site mutations. *ADAM23* represents an excellent candidate gene. It encodes a member of the disintegrin and metalloprotease domain (ADAM) family and belongs to a neuronal subfamily of ADAMs together with ADAM22 and ADAM11 [54]. ADAM23 binds two epilepsy-associated proteins, LGI1 and LGI2. *LGI1* is mutated in familial temporal lobe epilepsy-1 (ETL1) in humans, and *LGI2* is mutated in benign familial juvenile epilepsy (BFJE) in Lagotto Romagnolo dogs [33], [44]. The truncating mutations of *LGI1* or *LGI2* prevent their secretion and interaction with the ADAM23 complexes. The LGI1-ADAM23 complex is involved in the stimulation of neurite outgrowth and dendritic arborisation [55]. ADAM23 containing complex plays a role in pulling together both pre- and post-synaptic membranes, stabilizing and strengthening synaptic neurotransmission [56]. Furthermore, homozygous removal of *Adam23* from mice results in a seizure disorder and even heterozygous mice have lowered seizure thresholds [56]. We identified only a single non-synonymous variant from *ADAM23* gene, R387H, which is in strong LD with the associated intronic marker identified in the GWAS. The coding variant is highly associated and increases the epilepsy risk by 4-fold. However, given that the homozygous risk allele is common (22%) in controls, present frequently in 27 other breeds, and unlikely pathogenic, these results suggest that it is a polymorphism rather than a causative mutation. Based on our haplotype association analysis, the causative mutation is likely located in the same haplotype block tagged by these two variants. The fact that there were no changes in the expression of *ADAM23* in epileptic dogs suggests that the possible disease causing variant, if present in *ADAM23* at all, does not affect its transcript levels in the brain.

*KLF7* belongs to a large family of KLF transcription factors. *KLF7* is the only family member with a neuronally restricted expression during development. *KLF7* is required for neuronal morphogenesis and axon guidance in hippocampus, olfactory bulbs and cortex [45], [57]. We could not find any variants in the coding regions of *KLF7*. The identified locus contains also three other candidate genes, *DYTN*, *NDUFS1* and *FASTKD2*, that function in the CNS or have been associated with neuronal phenotypes. *DYTN* is poorly characterized but expressed in the CNS [58]. *NDUFS1* is a core component of the mitochondrial complex I system, and mutations in this system has been associated with neurodegenerative disorders [59]. *FASTKD2* is a cytochrome oxidase deficiency related gene and its mutations cause various neurological phenotypes including convulsions [60]. However, the phenotypes related to the latter two mitochondrial genes or systems are not restricted to CNS but affect other organs as well.

There are several possibilities where the actual predisposing mutation may be located. First, mutation may still lie in the regulatory regions of the *ADAM23* or *KLF7* genes. Second, mutation is present in the other candidate genes not screened yet. Third, we focused here only on the 1 Mb region showing strongest association, while our genotype data indicates a remarkable signal outside the most significant locus. This ∼2 Mb region contains also several candidate genes. To identify the causative variant we have initiated a targeted re-sequencing project to capture a 4 Mb locus on CFA37.

As part of our larger canine epilepsy research program we have collected samples from IE dogs in several breeds. To test the association of the CFA37 locus in other breeds, we screened the *ADAM23* intronic variant in eight additional breeds. We used Lagotto Romagnolos as a control breed in the study since we recently identified the causative mutation in the *LGI2* gene on CFA3 [33]. Our across breed analysis found some evidence for association in Kromfohrländer and Whippet breeds. However, a single marker association in a small sample cohort should be interpreted cautiously and confirmed with replication using more samples and markers.

The high prevalence of IE among BS dogs causes a severe health issue in the breed [37]. Although many dogs respond well to treatments, still almost every fifth epileptic dog is euthanized within three years after the onset [18]. There is a clear need for genetic counseling and for the development of marker-assisted breeding programs. Our study identifies a significant risk allele for IE. However, since the majority (75%) of the unaffected dogs also carries one or two copies of the risk allele, it cannot be used for efficient diagnostics. The identification of the causative mutation remains as an important task to improve breeding plans, to reveal a new candidate gene for human IEs, to identify novel IE pathways, and to establish the breed as a large therapeutic animal model for IEs. This study makes a breakthrough by mapping a novel IE locus and paves the way towards the discovery of the first mutation in the most common seizure type in dogs.

## Materials and Methods

### Study cohort

A cohort of Belgian Shepherd dogs including 159 epileptic cases and 148 unaffected controls collected in Finland (178 dogs), Denmark (65 dogs) and USA (64 dogs) was used in this study. The Finnish cohort included mainly Finnish dogs (64%) but also dogs from Sweden, Poland, Australia, Switzerland, Austria, Germany and the Netherlands. The Danish cohort has been described previously by Berendt *et al.* [7], [18] and the US cohort by Oberbauer *et al.* [35], [36] and the Finnish cohort in this paper (Table 1, Table S1). All study cohorts were collected through Breed Clubs, breeders and owners and epilepsy diagnoses were based on questionnaires, telephone interviews and clinical, neurological and para-clinical examinations on selection of dogs. The clinical characterization of the Finnish cohort was based on clinical examination on 17 affected dogs and 4 healthy controls from Finland and detailed owner-filled epilepsy questionnaires from 94 dogs (http://koirangeenit.fi/Tiedostot/EpilepsyQuestionnaire.doc). Epilepsy questionnaire requested information about the age of onset, the number, duration and frequency of seizures, anti-epileptic medication, and typical characteristics of the ictal, pre- and post-ictal phases of seizures.

Inclusion criteria for the case in all cohorts required that the dog had experienced at least two seizures. The age of onset was reported by the owners and was not used as exclusion criterion due to possible inaccuracies. The average age of onset was between 3.1 to 4.1 years in different cohorts (ranging from 3 months to 9 years in the Finnish cohort, from 1.5 years to 11 years in the Danish cohort and from 2 years to 5 years in the US cohort). None of the epileptic dogs were known to be affected by other diseases. The control dogs had no history of seizures and were over 7 years of age in all cohorts.

The cohort in the GWAS included selected dogs from the Finnish and Danish cohorts. The GWAS cohort did not include first degree relatives. The replication cohort was independent from GWAS and included samples collected in Finland, Denmark and USA. Fine mapping cohort contains samples collected in Finland and Denmark. It includes the GWAS samples and overlaps with replication study cohort (50 cases and 59 controls of the fine mapping study were included in the replication). The controls were matched to the cases according to country of origin and breed variant.

The Finnish Kennel Club's breeding database, KoiraNet, was utilized for Finnish pedigrees. EDTA-blood samples were collected for each dog with the owner's consent in the genetic analyses and genomic DNA was extracted using a commercially available kit (Puregene, Gentra Systems, Minneapolis, MN). We have a valid ethical permit (ESLH-2009-07827/Ym-23, expiring Oct 2012).

### Clinical studies

Clinical studies included clinical and neurological examination, blood and in selected cases CSF, MRI, EEG tests and were performed at the Referral Animal Neurology Hospital Aisti, Vantaa, Finland and Department of Small Animal Clinical Sciences, University of Copenhagen, Denmark. Blood examination included complete blood count and serum biochemistry (sodium, potassium, calcium, phosphorus, magnesium, glucose, total protein, albumin, globulin, cholesterol, blood urea nitrogen, creatinine, total bilirubin, alanine aminotransferase, aspartate aminotransferase, alkaline phosphatase, and creatine kinase). MRI examinations of Finnish dogs were performed as described previously [61]. CSF samples were collected from the cerebellomedullary cistern after MRI examination. Total cell count, cytology and protein concentration were evaluated.

EEG in Finnish dogs was performed under medetomidine sedation (0.04 mg/kg IM). An additional 0.02 mg/kg of medetomidine was given IM if the dog was not ready for examination 15 minutes after the initial injection. EEG examinations were performed in a quiet, darkened room. Dogs were placed in sternal recumbency and electrodes were placed transcutaneously in a 14 channel montage, modified from a 17-channel montage as described previously [62]. To assure good electrical contact with the electrodes, the scalp was defatted by rubbing vigorously with ethyl alcohol. Referential and bipolar montages (F7, F3, F4, F8, T3, C3, Cz, C4, T4, P3, Pz, P4, O1, O2 ;F4-C4, C4-T4, T4-P4, P4-O2, F3-C3, C3-T3, T3-P3, P3-O1, F4-F8, F8-T4, F3-F7, F7-T3, Cz-Pz, O2-Pz, Pz-O1; odd number = left hemisphere; even number = right hemisphere) were used. The acquisition parameters to record bio-electrical activity were set as follows: sensitivity = 5 µV/mm; time constant = 0.3 s; high filter (Hf) = 70 Hz; notch filter inserted; reference: on the bridge of the nose; ground: caudally to the external occipital protuberance; electrode impedance <3 KΩ; sampling rate 256 Hz. Sixteen EEG needles (thirty-gauge 15 mm monopolar stainless steel needle electrodes, Bionen S.a.S., Italy) were used as active, reference, and ground electrodes. No local infiltration of lidocaine was performed around electrode placement sites. Electrocardiogram and respiratory rates were recorded via polygraphic electrodes (EKG: sensitivity = 70 µV/mm, time constant = 0.1 s, Hf = 30 Hz; respiration - CHEST - : sensitivity = 20 µV/mm, time constant = 0.3 s, Hf = 30 Hz) connected to alligator clips (thin cable for bridge electrode, Bionen S.a.S., Firenze, Italy) and to a respiratory effort system (thoracic respiratory transducer, Bionen S.a.S., Firenze, Italy). EEG recording started when electrode placement was completed, and the total recording time was 20 min, including calibration and the initial impedance check. The EEG data were stored in the acquisition station (Halley Galileo, EBNeuro, Firenze, Italy) for later analysis.

### Genome-wide association analysis and fine-mapping

To map the epilepsy locus, 40 cases and 44 controls were genotyped using the Affymetrix Canine Genome 2.0 Array platinum set (Affymetrix, Santa Clara, CA, USA) containing 49,663 SNP markers. The genotyping was performed as a part of the LUPA project at the Centre National de Génotypage, Paris, France. The case-control association analysis was performed with PLINK v1.07 [63] with the criteria of MAF <0.05, call rate >75% and <25% of missing genotypes in individual dogs. After applying these filters 43,378 SNPs remained in the analysis for all dogs. Genome-wide significance was ascertained through 100 000 random permutations of the epilepsy phenotype. The genomic inflation factor for the population was 1.13 and the association p-values were adjusted for it.

Fine mapping and replication was performed with 96 selected SNPs from a 8.3 Mb region (12,660,614–20,989,289 bp) on CFA37. The SNP density was 1 SNP/100 Kb. All base pair positions mentioned in this article are based on CanFam 2.0. Genotyping was performed using the Sequenom (San Diego, CA, USA) iPLEX methodology at the Centre of Integrated Genomic Medical Research, University of Manchester, UK. A total of 201 samples were genotyped including samples from Finland (54 cases, 63 controls) Denmark (32 cases, 28 controls), Sweden (11 cases and 13 controls) and Middle Europe. After quality control (MAF <0.05, SNP call rate >0.75, individual call rate >0.75), a total of 58 SNPs and 83 cases and 99 controls were included in the association analysis with PLINK v1.07 using a single-marker association analysis and haplotype sliding window analysis with 3–5 markers at a time [63].

To confirm the nominal associations in the other chromosomes single SNPs on chromosomes 3 (BICF2P397912 at 82,766,251 bp), 4 (TIGRP2P58276 at 13,088,720 bp), 9 (BICF2P1288768 at 18,597,315 bp), 18 (TIGRP2P239410 at 15,846,321 bp) and 23 (BICF2G630382382 at 22,138,085 bp) were genotyped in 54 Belgian Shepherd cases and 62 controls in addition to the samples genotyped in the GWAS. The genotyping was performed using Custom Taqman SNP Genotyping Assays (Applied Biosystems by Life Technologies Corporation, Carlsbad, CA, USA). The polymerase chain reactions were performed according to the standard protocol provided by the manufacturer in 10 µl reaction volume, and run and analyzed using the Applied Biosystems 7500 Fast Real-Time PCR System (Foster City, CA, USA). The SNP genotype data was analyzed for association using PLINK v1.07 [63]. All the markers were in HWE (p>0.05) and the genotyping call rates ranged from 96–100%.

### Association in other breeds

A SNP located at 18,123,961 bp (BICF2P890779) on CFA37 was screened in 8 other breeds with epilepsy including Lagotto Romagnolo (23 cases, 23 controls), Miniature Pinscher (22 cases, 20 controls), Kromfohrländer (34 cases, 20 controls), Whippet (24 cases, 26 controls), Border Terrier (42 cases, 20 controls), Schipperke (63 cases, 48 controls), Finnish Spitz (62 cases, 81controls) and Finnish Lapphund (44 cases, 60 controls). The genotyping was performed using Custom Taqman SNP Genotyping Assays (Applied Biosystems by Life Technologies Corporation, Carlsbad, CA, USA) according to the standard protocol provided by the manufacturer in 4 µl reaction volume, and run and analyzed using the Applied Biosystems 7900HT Fast Real-Time PCR System (Foster City, CA, USA). The SNP genotype data was analyzed for association using PLINK v1.07. The genotyping call rates within breeds ranged from 91–100%, and there was no deviation from HWE in any of the breeds (p>0.05).

### Sequencing and mutation analysis

Exons and splice junctions were amplified by PCR with *ADAM23*-specific primers available upon request. The PCR products were purified with ExoSAP-IT kit (USB Corporation, Cleveland, Ohio) and sequenced with an ABI Prism 3730xl DNA analyzer (Applied Biosystems, Foster City, CA). The exon 12 variant (R387H) was sequenced in 155 BS cases and 111 controls and in three epileptic dogs in 38 other breeds. Pathogenicity of the mutation was predicted by programs Panther [64] and PolyPhen-2 [65].

### Gene expression analysis

Expression level of *ADAM23* was analyzed in three healthy Belgian Shepherds and three Belgian Shepherds with epilepsy. Samples from cortex cerebrum and cerebellum were taken immediately after euthanasia and snap-frozen in liquid nitrogen. Messenger RNA extraction, cDNA synthesis and quantitative PCR were performed as previously described [66]. RNA quality was ascertained and the RQI number determined by analysis on an Experion System (Bio-Rad, Hercules, CA, USA). Two primer sets for qPCR were designed; set 1: forward primer 5′-CCTGGCAGATGAAGACAACA, reverse primer 5′- GAGCCAAAGGCTTCAATCTG; set 2: forward primer 5′- AGCCACCTGCATCTGTGATT, reverse primer 5′- GTGCCCCCAAGGACAATAG. The gene *RPL4* was used as a reference gene in the expression level analysis. Expression data were analyzed using REST v2.0.7 [67].

## Supporting Information

Table S1 Summary of the clinical features of 94 epileptic Belgian Shepherds collected through the owner-filled epilepsy questionnaires.
(XLS)
